# An Adaptive Deep Ensemble Learning Method for Dynamic Evolving Diagnostic Task Scenarios

**DOI:** 10.3390/diagnostics11122288

**Published:** 2021-12-07

**Authors:** Kaixiang Su, Jiao Wu, Dongxiao Gu, Shanlin Yang, Shuyuan Deng, Aida K. Khakimova

**Affiliations:** 1 School of Management, Hefei University of Technology, Hefei 230009, China; 2018110745@mail.hfut.edu.cn (K.S.); yangsl@hfut.edu.cn (S.Y.); 2 School of Business, Northern Illinois University, DeKalb, IL 60115, USA; jwu3@niu.edu; 3 Key Laboratory of Process Optimization and Intelligent Decision-Making of Ministry of Education, Hefei 230009, China; 4 SpotHero, Chicago, IL 60603, USA; lance0108@gmail.com; 5 Scientific-Research Center for Physical-Technical Informatics, Russian New University, Radio St., 22, 105005 Moscow, Russia; aida_khatif@mail.ru

**Keywords:** adaptive deep ensemble learning, dynamic evolving diagnosis, intelligent health knowledge discovery, personalized health management

## Abstract

Increasingly, machine learning methods have been applied to aid in diagnosis with good results. However, some complex models can confuse physicians because they are difficult to understand, while data differences across diagnostic tasks and institutions can cause model performance fluctuations. To address this challenge, we combined the Deep Ensemble Model (DEM) and tree-structured Parzen Estimator (TPE) and proposed an adaptive deep ensemble learning method (TPE-DEM) for dynamic evolving diagnostic task scenarios. Different from previous research that focuses on achieving better performance with a fixed structure model, our proposed model uses TPE to efficiently aggregate simple models more easily understood by physicians and require less training data. In addition, our proposed model can choose the optimal number of layers for the model and the type and number of basic learners to achieve the best performance in different diagnostic task scenarios based on the data distribution and characteristics of the current diagnostic task. We tested our model on one dataset constructed with a partner hospital and five UCI public datasets with different characteristics and volumes based on various diagnostic tasks. Our performance evaluation results show that our proposed model outperforms other baseline models on different datasets. Our study provides a novel approach for simple and understandable machine learning models in tasks with variable datasets and feature sets, and the findings have important implications for the application of machine learning models in computer-aided diagnosis.

## 1. Introduction

Many different factors are often taken into account when diagnosing a disease. The complexity of the disease (such as the risk levels associated with multiple diseases) and the diagnostic knowledge available to the physician [1,2] can influence the correct diagnosis of the disease [3]. These complicated factors have raised many challenges for medical professionals, especially those who are young and inexperienced [4]. Machine learning is widely adopted to develop medical auxiliary diagnostic systems [5], which are also known as Computer-Aided Diagnosis (CAD) systems. CAD systems are important tools that provide disease diagnosis and prognosis [6,7]. They do not only help doctors make quick decisions and save patients’ time but also reduce the uncomfortable experience of patients by replacing invasive approaches [8]. CAD systems use a wide spectrum of machine learning methods [9], ranging from single prediction models such as Support Vector Machine (SVM) and Decision Tree (DT), to ensemble and deep learning models, such as Random Forest (RF), Extreme Gradient Boosting (XGBoost) and Deep Neural Network (DNN).

When CAD is used to assist diagnosis, effective feature engineering can be realized with the help of doctors, which makes it possible for some classical machine learning methods with better understanding to achieve better performance than deep learning models [10]. Appropriate features can be obtained through feature selection algorithms [11], selection methods based on physician experience [10], or other methods. On the other hand, many models based on deep neural networks may hinder the efficiency of the interaction between doctors and the system due to the incomprehensible nature of its decision-making process [12,13], while highly complex models are also not conducive to the physician’s adjustment to reduce diagnostic bias [14,15]. Therefore, improving the performance of relatively simple models with high comprehensiveness (such as accuracy and generalization in the face of changing data) remains important for CAD [16].

Ensemble learning is a class of methods that utilize more than one machine learning model to improve prediction results [17]. The performance of an ensemble learning model integrating the results of individual models (i.e., base learners) is usually better than that of the individual models [18]. For instance, Tseng [19] integrated five machine learning classifiers to propose an ensemble model for diagnosing recurrent ovarian cancer. Ensemble learning usually selects an optimal set of base learners and then combine them using a specific fusion method. Thus, the decision on choosing base learners and integrating them is critical. To ensure optimal performance, the base learners should have both good performances and enough diversity [20]. To aggregate base learners, classifier fusion methods are typically used. Such methods may include majority voting, support function fusion, and stacking [21].

The optimal set of base learners and fusion method may change when an ensemble learning model is applied to different datasets [22]. Due to the heterogeneity of datasets and the diversity of disease types, a fixed algorithm structure is likely to limit the accuracy of diagnosis. Prior research has proposed different strategies to make an ensemble model generalizable to different problems. For instance, Al-Tashi [23] used wavelet transformation and singular value decomposition to reduce feature space dimensions. This method relies on the projection of features instead of specific features, which improves model generalization on diagnostic performance. Yet, similar to linear models, this approach still focuses on reducing model complexity rather than making the model adaptive to different problems and datasets. Zhou [24] experimented with a deep forest ensemble architecture that consists of two kinds of random forest algorithms. However, adopting a fixed number and type of classifier will still hinder the performance of the system in the face of different problems.

Previous studies have made good progress in adapting ensemble models for heterogeneous problems. However, most of them adopt a fixed structure, which can only ensure that the performance of the model remains relatively stable, but they do not help the model achieve optimal performance across different diagnostic tasks, changing datasets, and diagnostic features. Specifically, in some real-world assisted diagnosis scenarios, training datasets will have significantly different volumes and features depending on different diagnostic tasks and different hospitals [22]. Diagnostic data are still difficult to share as an important asset for hospitals, which means that it is difficult for small hospitals to obtain large amounts of data sufficient to support the training of complex deep models, so it is important that the auxiliary diagnostic models can maintain good performance against small datasets, and the performance of the models needs to be robust in the face of different features of different diagnostic scenarios. Therefore, designing and constructing an adaptive deep ensemble learning method for simple base learners with high understandability can further improve the accuracy, reproducibility and interpretability of the deep ensemble learning model and promote its wider application in the field of bioinformatics and CAD [25].

In this study, we propose a DEM based on a Tree-Structured Parzen Estimator (TPE) to address the above problems. DEM is a class of deep learning model based on cascade forest structure. Different from traditional deep neural networks, each layer of DEM is composed of base classifiers. In this study, we use TPE to optimize the number of base classifiers per layer so that it can dynamically adjust the number of base classifiers when applied to different datasets. The TPE method has been widely used for optimizing hyperparameters [26]. We further use four advanced ensemble learners to form a base classifier pool. This ensures that the base learners have good diversity, which is critical to ensemble learning [27]. The four ensemble learners are Random Forest (RF), Extra Trees (ET), AdaBoost, and Gradient Boosting Decision Tree (GBDT). By dynamically adjusting the system structure based on data, the proposed algorithm can dynamically search for optimal solutions when applied to different problems.

Overall, our model uses TPE for classifier selection and DEM for classifiers fusion. The proposed model has three main advantages:

(1) Our proposed model is based on the integration of simple and comprehensible models. Therefore, this model needs to learn fewer parameters than the deep neural network-based model and therefore requires less training data while being more easily accepted and understood by physicians in practical applications.
(2) Our proposed model can dynamically adjust its structure to maintain good performance in tasks with different datasets and feature sets.
(3) Our proposed model can be flexibly tuned for continuous optimization, e.g., future studies for base classifiers can enable the overall performance of the model.

To examine the performance of the TPE-DEM model compared with other benchmark models, we conducted validation experiments on six datasets with significant differences (the differences are reflected in the different volumes, number of features, and the proportion of negative and positive data). We first use two different datasets representing different diagnostic tasks and describe the optimal hyperparameters and performance of the proposed model on two datasets. The first is breast cancer diagnostic data from our partner hospitals, and the second is the coronary artery disease prediction dataset from the UCI public datasets. Then, to further validate the performance of the proposed model on different datasets, we used four additional UCI public datasets for evaluation experiments. The first two datasets are oriented to medical diagnosis tasks. The last two datasets are oriented to tasks in other scenarios, where the last dataset has a significantly higher volume than the others. Our experimental results demonstrate that the proposed model has good performance on small volume datasets. However, as a deep model, its performance on datasets with large volumes is more outstanding than other benchmark models.

The remainder of the paper is organized as follows. Section 2 reviews previous studies and their relevance to our study. Section 3 describes the proposed TPE-DEM model, and Section 4 introduces the six datasets and metrics that we used to evaluate the model. In Section 5, we analyze the experimental results and discuss the theoretical and practical implications of our research. In the final section, we summarize our research and point out limitations that still need to be addressed in the future.

## 2. Related Works

Ensemble learning techniques combine multiple base learners and can obtain better prediction performance than single learners. Bagging, boosting, and stacking are the most common ensemble approaches. Bagging combines the predictions of individual base learners by voting. Boosting iteratively constructs new models based on the prediction error of previous models. Stacking trains a meta learner using the predictions of individual base learners. The meta learner determines the weights of the predictions in a supervised fashion. The construction of an ensemble model mainly involves approaches for generating (of a pool of classifier), selecting (categories and quantities of classifiers) and integrating (the prediction results of each classifier to generate the final output) [28].

Chandra [29] suggests that the most promising direction is to generate a pool of accurate and diverse algorithms. Therefore, the optimal ensemble model should combine base learners with good individual performance and enough level of diversity. The selection stage in ensemble learning determines the type and number of base learners. The selection strategy can be static or dynamic [30]. The static strategy combines base learners regardless of data, while dynamic selection chooses the most appropriate base learners for a given dataset. Existing research has extensively studied algorithms for finding an accurate and diverse set of base learners for ensemble learning. For instance, Brun [31] proposed a dynamic classifier selection framework and demonstrated through experiments that training different classifiers based on different problems and datasets can improve classification accuracy. Junior [32] proposed a reduced minority k-nearest neighbors method based on k-nearest neighbors, which effectively solves the problem of prediction bias caused by unbalanced data in a credit score prediction task. Previous studies have proved that dynamic classifier selection and the combination can improve the performance of classifiers facing different data types and different scenarios, and our study also proves this theory. However, differently from previous work, the model we proposed turns the classifier selection problem into an optimization problem, making the process of classifier selection more rapid and further improving the performance of the ensemble model by combining it with DEM [32].

Many search algorithms have been considered for optimization, such as Genetic Algorithms (GA) [33] and Evolutionary Algorithms (EA) [34]. The major limitation of these methods is that they often use a significant amount of time to evaluate hyperparameters. Gaussian process-expected improvement [35] and Gaussian process-predictive entropy search [36] methods use Gaussian Process (GP) to estimate the error caused by different hyperparameters. These methods employ Expected Improvement (EI) and predictive entropy search acquisition functions. Although GP is simple and flexible, its covariance matrix processing needs a lot of computation [37]. Researchers proposed TPE, which now has been widely used for hyperparameter optimization. Recent work has also used TPE to optimize the hyperparameters of convolutional neural networks to improve the performance of the model in the lung nodule recognition task [38]. In this paper, our proposed model needs to dynamically adjust the hyperparameters for better performance in the face of different diagnostic tasks, but the optimal computation of hyperparameters entails additional time loss. To minimize the time loss, our model requires a faster optimization algorithm. Compared with other optimization algorithms, TPE can complete the optimization task in less time; therefore, we choose TPE as the hyperparameter selection method in this paper.

The integration strategy of the ensemble learning model often depends on the specific situation. Each base learner can have equal or different weights, and the integration strategy usually affects the accuracy of the final model [39]. The rule for combining base learners could be supervised or unsupervised. Sum and majority voting are well-known unsupervised methods. Stacking is a supervised method. The predicted results from each base learner are merged into new features and trained using the meta learner [40]. Recently, researchers have introduced mechanisms to combine ensemble learning methods and various deep learning algorithms to enhance prediction performance. Zhou [24] proposed a cascade forest ensemble based on gcForest for better representation learning. In this model, based on the deep neural network model, the author replaced each neuron with a tree-based classifier. In general, the performance of traditional deep forest ensemble models based on the static integration method will be greatly affected by the change in data. Based on the traditional deep forest ensemble scheme, we use the TPE method to optimize the structure of the model to dynamically adjust the type and number of base learners in the model according to different datasets. Experiments show that the method we propose in this study has better performance than popular baselines and maintains stable performance on different datasets. We further evaluate the new system in diseases diagnosis.

## 3. Methods

### 3.1. Model Design

**Figure 1:**
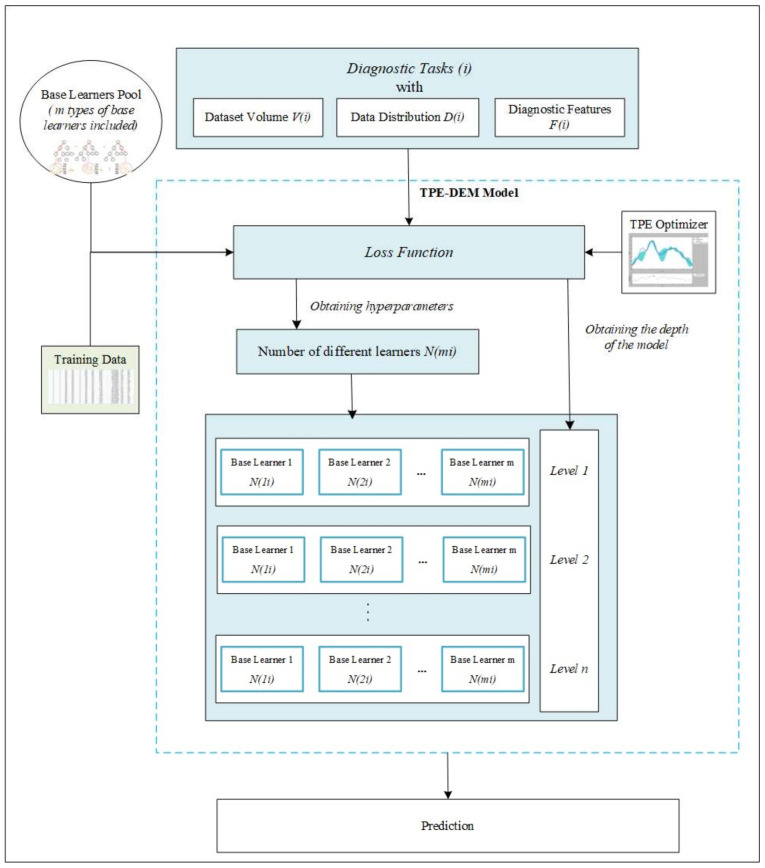
Framework of our proposed methods.

To address the challenges in data-driven medical diagnosis, where data are complex and heterogeneous, this study proposes a novel multiple classifier system that uses TPE for the selection and DEM for the integration of base learners. The general framework of the proposed algorithm is illustrated in Figure 1. The DEM component inherits the advantages of the Cascade Forest Structure proposed by [24]. It works like a neural network model by learning the information in the data through layers. These derived features, combined with the original features, are then passed over. It automatically increases the depth until the testing set accuracy is no longer improved. Majority voting is performed on each layer until the last layer obtains the final results. As shown in Figure 1, we extend the Cascade Forest Structure proposed by [24] by optimizing the selection of base learners using a pool of highly diverse candidates. This paper takes the number of different base classifiers included in the proposed model when facing different task scenarios as hyperparameters. Our proposed model obtains the optimal hyperparameters by using the TPE method. Thus, the user does not need to spend a lot of effort adjusting the hyperparameters to optimize the model. The proposed method has achieved superior performance in our experiments. Although it is a deep model, the number of parameters based on tree structure is much smaller than that of a typical deep neural network. Thus, less training data are required.

For the pool of base learners, we use Random Forest (RF), ExtraTree (ET), AdaBoost, and Gradient Boosting Decision Tree (GBDT). All of these are powerful ensemble methods themselves (we introduce these base learners briefly in the next section). Using a combination of these four base learners can ensure both accuracy and diversity of the pool. This is essentially different from the Cascade Forest structure, which used only random forests. In our proposed algorithm, each classifier $m_{i}$ predicts an estimated class distribution $p_{i}$. We optimize the number of base learners by minimizing a loss function given by the average outputs of all of the classifiers. We predict the class labels based on the predicted probabilities *p* for classifier and the class label $\hat{y}$ via majority voting of each classifier $m_{i}$. Assuming the example as a binary classification task with class labels *k*∈{0,1}, it can be expressed as follows:

(1) $$\hat{y}=arg\;\underset{k}{max}\sum_{m_{i\in \theta }}\overset{w_{j}}{\underset{j=0}{\sum }}p_{kij}$$

where $p_{kij}$ represents the probability that the *j*th $m_{i}$ classifier predicts that the current label is *K*. Note that $m_{i}$ in this study belongs to the pool θ of four basic ensemble learners described above, while in other task scenarios, θ can be composed of other different base learners in different scenarios.

Based on Equation (1), we minimize the majority voting error between the true label and the predicted label. The number of classifier $m_{i}$ is denoted as $w_{i}$ and $w_{i}$ ∈ N = {0, 1, 2, 3, …}. When the value of $w_{i}$ is 0, classifier $m_{i}$ is not selected.

### 3.2. Base Learners

We use four common ensemble models with a proven excellent performance to build the base learner pool because ensemble models generally perform better than individual models in many machine learning tasks and are more stable in the face of unbalanced data sets [41,42]. In addition, the decision-making process of these models based on a decision tree is easier to be understood by doctors than those based on neural networks.

(1) ET is a tree-based ensemble learning model that strongly randomizes attributes and split points. It simultaneously splits new nodes to maintain strong randomness among the base decision trees [43]. Based on the integration of many base classifiers with strong randomness, ET often has excellent performance.
(2) GBDT is a popular model proposed by Friedman [44]. This model consists of multiple decision trees. The results of all the trees are added together to make the final prediction. GBDT makes each base learner fit the residual of the previous learner iteratively to reduce final prediction errors.
(3) RF trains a fixed number of weak decision trees using randomly selected training samples and uses the results of these trees to generate final predictions by voting. Random forest rarely overfits and is robust to noise in the data [45].
(4) AdaBoost is a classical ensemble learning algorithm. It combines several weak learners into strong learners. It iteratively assigns more weights to the samples mispredicted by the previous weak learners. New learners are subsequently trained on these samples [46].

Note that since the base classifier chosen for this paper is based on a tree model, we performed data preprocessing before the data were fed into the model. Specifically, when a dataset contains both real and categorical values, the real values are discretized. For example, we divide the age attributes into three categories according to (0, 30), [30, 60) and [60, ∞), and doctors can incorporate their experience into the system by changing the interval division in practical applications. Future research that attempts to use other types of models as base classifiers could also use encoding methods such as one-hot to process the data as input to the model.

### 3.3. Model Optimization Based on TPE

In our study, TPE was used to determine the number of base learners by optimizing the loss function because of its superior convergence and exploration capabilities. When training a supervised learning algorithm, it is often necessary to find a set of hyperparameters that can make the model performance reach its peak. Bayesian optimization is one of the practical ways for hyper-parameter optimization. In its essence, Bayesian hyperparameter optimization selects hyperparameters based on probability. Sequential Model-Based Optimization (SMBO) methods [47] are a type of Bayesian optimization. This method attempts to obtain better hyperparameters by continuously using Bayesian reasoning and updating probabilistic models. There are five aspects of model-based hyperparameter optimization:

1. A domain of hyperparameters over which to search.
2. An objective function that can be optimized to obtain the corresponding score by optimizing the hyperparameters.
3. The surrogate model of the objective function.
4. A criterion, called a selection function, for evaluating which hyperparameters could be chosen in the next step based on the surrogate model.
5. A history consisting of (score, hyperparameter) pairs used by the algorithm to update the surrogate model.

Several different methods are derived based on SMBO, which construct proxies and select hyperparameters using different rules. Several common choices for the surrogate model are GP, random forest regressions, and TPE. We focus on TPE in this paper. TPE is a nonstandard Bayesian-based optimization algorithm that models error distribution nonparametrically [26]. TPE creates $l\left(x\right)$ and *g*(*x*) as two hierarchical processes to generate all domain variables. These processes model the domain variables when the objective function is below and above a specified quantile $y^{*}$. Specifically, TPE models *p*(*x*|*y*) by transforming the generative process. The benefit of using TPE is that it naturally supports domains with specified conditional variables.

(2) $$p\left(x|y\right)=\left\{\begin{array}{c} l\left(x\right)\;if\;y<y^{*}\\ g\left(x\right)\;if\;y\geq y^{*} \end{array}\right.$$

where $l\left(x\right)$ is the density estimated from the observations {$x^{i}$} such that the corresponding loss *f*($x^{i}$) is less than $y^{*}$. $g\left(x\right)$ is the density estimated from the remaining observations.

In particular, the method that we proposed turns the classifier selection problem into a hyperparameter optimization problem—it searches for the optimal number of classifiers. In the process of model construction, the method iteratively minimizes a loss function by selecting a different number of classifiers. In each iteration, the TPE will obtain the range that is most likely to produce the best hyperparameter based on the current hyperparameter and the current loss and then apply the best range in the next iteration. This method will greatly reduce the number of iterations and model training time.

## 4. Evaluation

### 4.1. Datasets

In this work, we used six different datasets to examine our proposed model—the first dataset from our collaborating hospital and the remaining five datasets from the UCI dataset. We first present an overview of all datasets (see Table 1). In this paper, we consider the experiments based on two datasets as two different diagnostic tasks and use them as examples for the demonstration of the model workflow, so we describe in detail the first two datasets and the experimental procedure based on the first two datasets. We also provide the model’s results on the remaining four datasets compared with other models to further validate the performance of the proposed model. The first dataset was used to predict breast cancer and was processed by senior physicians from a collaborating grade-A3 (the highest grade for hospitals in China) hospital in eastern China. The dataset contains 10 of the most common features from clinical and regular examinations identified by physicians (see Table 2). In this dataset, patients’ conditions are divided into two categories: benign (negative) or malignant (positive).

The second dataset was used to predict whether a patient has coronary artery disease. This dataset is the Z-Alizadeh Sani dataset obtained from the UCI dataset [48]. The dataset contains information about 303 patients, 216 of which suffered from coronary artery disease. A total of 54 features were collected from each patient. These features come from different data sources, including patients’ demographics, symptoms, physical examination results, electrocardiography, echocardiography, and laboratory tests (see Table 3). In this dataset, patients’ conditions are divided into two categories: negative or positive.

### 4.2. Baselines and Metrics

In order to show the effectiveness of the proposed system, we selected six baselines for comparison, including RF, AdaBoost, ET, GBDT, TPE-Voting and DEM. Random forest, AdaBoost, ExtraTrees, and GBDT are the current ensemble learning models with good performance. TPE-Voting is an ensemble learning model which uses TPE method to optimize the voting weight in the integration process. DEM is a traditional deep forest model with a fixed structure. Using these baselines, we can compare the performance of TPE-DEM to that of traditional ensemble learning models and deep forest models to show the advantages of TPE-DEM model.

We measure the performance of our model using a number of metrics that are recognized by a wide range of work [51]. The prediction metrics used are precision, F-measure, accuracy and Area Under the Receiver Operating Characteristic (AUC). They are defined as follows:

(3) $$Precision=\frac{TP}{TP+FP}$$

(4) $$F-measure=2\times \frac{Precision\times Recall}{Precision+Recall}$$

(5) $$Accuracy=\frac{TP+TN}{TP+FP+TN+FN}$$

where *TP*, *TN*, *FP*, and *FN* denote the numbers of true positives (hits), true negatives, false positives (false alarms), and false negatives (misses), respectively. The Receiver Operating Characteristic (ROC) curve is an effective method for assessing the performance of a model over all possible thresholds. AUC is the area under the ROC curve, and it is the most commonly used summary measure of a ROC curve [52].

### 4.3. Experimental Procedure

We conducted experiments on six corresponding datasets, and a 10-fold cross-validation approach was used to evaluate our proposed algorithm. Both datasets are randomly divided into ten stratified subsamples of equal size. For each fold, nine subsamples are used to train the model, and the rest are used for testing. Each trial is run ten times. The results are averaged across the 100 runs. We also use paired t-test to test if models differ significantly in performance. All of the classifiers are implemented using the Scikit-learn Python library [53] with default parameters, except that the TPE algorithm is based on a Python tool named hyperopt [54].

## 5. Results and Discussion

### 5.1. Performance of TPE-DEM

In the breast cancer prediction task (Breast Cancer Prediction dataset), the TPE algorithm obtained the classifier value [2, 5, 5, 0], corresponding to 2 random forests, 5 ExtraTrees, 5 AdaBoost, and 0 GBDT are the optimal hyperparameters of our proposed model in the current task. The model performance is optimal when the optimal hyperparameters are used, so in Table 4, we use the model performance based on the optimal hyperparameters as the performance of TPE-DEM in the current task. In addition to the proposed TPE-DEM, we also tested other methods, including each base ensemble classifier, TPE with majority voting, and DEM without TPE for selection which uses all four base classifiers. TPE-DEM performs better than the other classifiers in both accuracy and F-measure. The performance of TPE-DEM is consistently superior to other baselines.

In the coronary artery disease prediction task (Z-Alizadeh Sani dataset), we initially used XGBoost for feature selection to reduce overfitting and computational complexity. We then selected 28 features with coefficients greater than 0.01 as the new input. The TPE algorithm obtains the weight value [3, 3, 1, 0], corresponding to 3 RF, 3 ET, 1 AdaBoost, and 0 GBDT are the optimal hyperparameters of our proposed model in the current task. The model performance is optimal when the optimal hyperparameters are used, so in Table 5, we use the model performance based on the optimal hyperparameters as the performance of TPE-DEM in the current task. Overall, TPE-DEM outperforms all other classifiers.

To further demonstrate the performance of the proposed model, we also show the experimental results of the model on the Indian liver patient dataset (see Table 6), Breast Cancer Wisconsin dataset (see Table 7), Cervical Cancer dataset (see Table 8), and Thyroid Disease dataset (see Table 9). The results demonstrate that some of the baseline models while achieving better performance on some datasets have substantially lower performance on specific datasets. However, our proposed model can maintain stable and good performance in different datasets.

### 5.2. Discussion

Many machine learning or deep learning models are now being applied to assist diagnostic tasks to help physicians make diagnostic decisions. However, in practical applications, physicians need to give their judgments supported by sufficient evidence, so the understandability of the models in CAD tasks is crucial. This paper proposes a novel DEM that integrates several simple and easily understandable models and dynamically adjusts the structure to maintain stable performance across different CAD tasks. Our experiments on six datasets demonstrate that our proposed TPE-DEM model can further improve the simple model’s performance and obtain good performance on datasets with different volumes and features.

Our study also contributes to the ensemble learning literature. Ensemble models usually have better prediction accuracy than individual base learners. However, popular ensemble models often use a fixed model structure in terms of a number of base learners and a number of integration layers. This potentially limits their ability to adapt to different problem domains. Using TPE, our proposed TPE-DEM model automatically found the optimal numbers of base learners and integration layers. Our experimental results on six different datasets prove that the model we proposed achieves effective integration of the base learner on different datasets, and TPE-DEM has better performance under multiple evaluation metrics. It is worth noting that in this work, we built a pool containing four base learners in order to select the base learners. In practical applications, more different and advanced base learners can be included in the pool to better cope with different tasks.

Practically, TPE-DEM does not require much intervention from human experts, which benefits medical professionals by allowing them to use a single type of model for a variety of diagnosis tasks. This reduces the complexity of a medical information system, making it easier to maintain and upgrade [55]. At the same time, as the models based on deep neural networks have not been able to effectively raise the interpretation to the understanding of the end user [56,57], too-complex models will hinder doctors’ trust in CAD systems [12,13]. In our work, the integration of relatively simple models (such as tree-based models) ensures the system performance while taking into account doctors’ understanding of the model decision-making process. Therefore, this study has important implications for the practical application of CAD systems.

## 6. Conclusions

In this paper, we proposed a TPE-DEM model based on the traditional DEM model. Our proposed model transforms the process of integrating different simple base learners into an optimization problem by using a TPE optimization algorithm to obtain the optimal hyperparameters of the model for various diagnostic tasks. Due to the integration of simple models, our proposed model requires less training data and is more easily understood by physicians than deep neural network-based models. When faced with different diagnostic tasks and datasets, our proposed model can change its structure by dynamically adjusting hyperparameters to maintain good performance in various tasks.

To evaluate the effectiveness of TPE-DEM, we validated its performance on six different datasets. The first and fourth datasets have good features and more balanced data distribution. The experimental results show that TPE-DEM and other baseline models can effectively learn from the data and achieve good performance. However, TPE-DEM performs on average 2% higher than other baseline models in all four metrics on the first dataset and 1% higher than other baseline models in three metrics on average on the fourth dataset for TPE-DEM. When the datasets are somewhat unbalanced (the second and third datasets), the performance of all models decreases. Still, TPE-DEM outperforms the rest of the baseline by more than 1.5% on average for all four metrics. In the experiment based on the fifth dataset, Precision and F-measure metrics were significantly lower for all models affected by the dataset. However, TPE-DEM outperformed the other baseline models by more than 6% on average in these two metrics. Overall, TPE-DEM outperforms the other baseline models on all six datasets. The advantage of TPE-DEM is more pronounced when deficiencies in the dataset degrade the performance of all models.

However, the proposed algorithm is not without limitations. For example, the algorithm specifies that the classifiers and their number must be the same in each layer of the deep ensemble structure. Additionally, some recent studies proposed other types of classifier selection algorithms. In our experiments, we did not implement these algorithms for our testing datasets due to the lack of specific details. Thus, although we have directly compared our proposed method to some very competitive baselines, we have not obtained the results of these recent algorithms using our testbed. Future research may contribute to this field through a comprehensive benchmarking of different classifier selection algorithms and identify state-of-the-art. Further research may also analyze the theoretical performance of TPE-DEM.

## Figures and Tables

**Table 1:** Overview of the six datasets.

Dataset Name	Volume	Distribution	Number of Features
Breast Cancer Prediction	334	170 positive and 164 negative	10
Z-Alizadeh Sani	303	216 positive and 87 negative	54
Indian Liver Patient [49]	583	416 positive and 167 negative	10
Breast Cancer Wisconsin [49]	569	212 positive and 357 negative	32
Cervical Cancer [49]	858	55 positive and 803 negative	36
Thyroid Disease [50]	7200	6644 positive and 556 negative	21

**Table 2:** Features of Breast Cancer Prediction.

Attribute	Type	Description of Attribute
Age	Continuous	Patient’s age
Location	Discrete	Location of the patient’s mass
Node	Continuous	Number of metastatic lymph nodes
Density	Discrete	Density of the patient’s mass
Clarity	Discrete	Clarity of the patient’s mass margin
Area	Continuous	Area of the patient’s mass
Regulation	Discrete	Regulation of the patient’s mass border
Surface Smoothness	Discrete	Smoothness of the patient’s mass surface
Nipple	Discrete	Whether a woman with breast tumor has nipple discharge
Family_History	Discrete	Whether the patient has a family history of breast cancer

**Table 3:** Features of Z-Alizadeh Sani dataset.

Feature Type	Feature Name	Data Type
Demographic	Age	Real number
	Weight	Real number
	Sex	Categorical
	Length	Real number
	Body mass index	Real number
	Diabetes mellitus	Categorical
	Hypertension	Categorical
	Current smoker	Categorical
	Ex-smoker	Categorical
	Family history	Categorical
	Obesity	Categorical
	Chronic renal failure	Categorical
	Cerebrovascular accident	Categorical
	Airway disease	Categorical
	Thyroid disease	Categorical
	Congestive heart failure	Categorical
	Dyslipidemia	Categorical
Symptom and examination Density	Blood pressure (mm Hg)	Real number
	Pulse rate (ppm)	Real number
	Edema	Categorical
	Weak peripheral pulse	Categorical
	Lung rales	Categorical
	Systolic murmur	Categorical
	Diastolic murmur	Categorical
	Typical chest pain	Categorical
	Dyspnea	Categorical
	Function class	Real number
	Atypical	Categorical
	Nonanginal chest pain	Categorical
	Exertional chest pain	Categorical
	Low-threshold angina	Categorical
ECG	Rhythm	Categorical
	Q wave	Categorical
	ST depression	Categorical
	T inversion	Categorical
	Left ventricular hypertrophy	Categorical
	Poor R-wave progression	Categorical
Laboratory and echo	Fasting blood sugar (mg/dL)	Real number
	Creatine (mg/dL)	Real number
	Triglyceride (mg/dL)	Real number
	Low-density lipoprotein (mg/dL)	Real number
	High-density lipoprotein (mg/dL)	Real number
	Blood urea nitrogen (mg/dL)	Real number
	Erythrocyte sedimentation rate (mm/h)	Real number
	Hemoglobin (g/dL)	Real number
	K (mEq/lit)	Real number
	Na (mEq/lit)	Real number
	White blood cell (cells/mL)	Real number
	Lymphocyte (%)	Real number
	Neutrophil (%)	Real number
	Platelet (1000/mL)	Real number
	Ejection fraction (%)	Real number
	Region with RWMA	Real number
	Valvular heart disease	Categorical

**Table 4:** Results of comparison with classification models (Breast Cancer Prediction dataset).

	Precision	F-Measure	Accuracy	AUC
Random Forest	91.83%	89.49%	89.58%	95.04%
AdaBoost	84.07% *	83.45% *	83.30% *	91.85% *
ExtraTrees	88.73% *	84.95% *	85.33% *	92.80%
GBDT	92.81%	89.69%	89.92%	95.24%
TPE-Voting	87.57% *	86.52% *	86.42% *	94.04% *
DEM	92.79% *	88.01% *	88.51% *	94.93% *
TPE-DEM	95.36%	90.91%	91.26%	96.08%

* *p*-values are significant at α = 0.05.

**Table 5:** Results of comparison with classification models (Z-Alizadeh Sani dataset).

	Precision	F-Measure	Accuracy	AUC
Random Forest	88.86%	91.14%	86.95%	92.72%
AdaBoost	87.88% *	88.94% *	84.07% *	88.05% *
ExtraTrees	90.88% *	90.35% *	86.33% *	90.83% *
GBDT	90.02%	91.84%	88.05%	92.45%
TPE-Voting	90.05% *	90.51% *	86.33% *	91.55% *
DEM	89.11% *	90.12% *	85.73% *	91.84% *
TPE-DEM	91.03%	92.76%	89.43%	92.99%

* *p*-values are significant at α = 0.05.

**Table 6:** Results of comparison with classification models (Indian Liver Patient dataset).

	Precision	F-Measure	Accuracy	AUC
Random Forest	87.04% *	73.84% *	90.15%	71.46% *
AdaBoost	78.30% *	72.08% *	86.65% *	71.36%
ExtraTrees	85.40% *	75.62%	89.63%	71.53%
GBDT	85.33% *	73.71% *	89.34%	69.53%
TPE-Voting	85.86%	74.03%	90.15%	73.21%
DEM	82.47%	73.16%	85.44%	73.21%
TPE-DEM	87.11%	75.48%	90.44%	75.22%

* *p*-values are significant at α = 0.05.

**Table 7:** Results of comparison with classification models (Breast Cancer Wisconsin dataset).

	Precision	F-Measure	Accuracy	AUC
Random Forest	96.18%	94.67%	96.14%	98.11%
AdaBoost	96.30%	94.77% *	96.13% *	98.12% *
ExtraTrees	97.16%	95.86%	97.01%	98.15%
GBDT	95.80% *	94.52% **	95.96% *	98.33% *
TPE-Voting	94.34%	92.79%	94.73%	98.36%
DEM	97.59%	95.42%	97.02%	98.36%
TPE-DEM	97.63%	95.90%	97.35%	98.38%

* *p*-values are significant at α = 0.05. ** *p*-values are significant at α = 0.01.

**Table 8:** Results of comparison with classification models (Cervical Cancer dataset).

	Precision	F-Measure	Accuracy	AUC
Random Forest	71.45%	59.72% *	95.46%	97.00%
AdaBoost	58.95% *	50.93% **	94.18% *	88.21% *
ExtraTrees	68.67% *	64.47% *	95.46%	95.46%
GBDT	70.50%	66.36%	95.81%	96.01%
TPE-Voting	62.17% *	59.16% *	94.87%	92.90% *
DEM	70.04% *	64.82% *	95.34%	92.90% *
TPE-DEM	76.02%	67.02%	95.58%	97.01% *

* *p*-values are significant at α = 0.05. ** *p*-values are significant at α = 0.01.

**Table 9:** Results of comparison with classification models (Thyroid Disease dataset).

	Precision	F-Measure	Accuracy	AUC
Random Forest	99.83%	99.76%	99.55%	98.92%
AdaBoost	99.66%	99.74%	99.52%	98.31%
ExtraTrees	98.11% *	98.96%	98.06% *	98.80%
GBDT	99.83%	99.76%	99.55%	98.92%
TPE-Voting	96.36% *	97.94% *	96.13% *	97.80% *
DEM	98.22%	98.97%	98.09% *	97.80%
TPE-DEM	99.86%	99.81%	99.66%	98.94%

* *p*-values are significant at α = 0.05.

## Data Availability

This paper contains 6 datasets, the first dataset presented in this study are available on request from the corresponding author and the data are not publicly available due to data attribution rights. The rest datasets presented in this study are openly available in UC Irvine Machine Learning Repository at http://archive.ics.uci.edu/ml/index.php, reference number [49].

## References

[1] Gómez-Vallejo H.J., Uriel-Latorre B., Sande-Meijide M., Villamarín-Bello B., Pavón R., Fdez-Riverola F., Glez-Peña D. (2016). A case-based reasoning system for aiding detection and classification of nosocomial infections. Decis. Support Syst..

[2] Pee L.G., Pan S.L., Cui L.L. (2019). Artificial intelligence in healthcare robots: A social informatics study of knowledge embodiment. J. Assoc. Inf. Sci. Technol..

[3] Lin Y.K., Chen H.C., Brown R.A., Li S.H., Yang H.J. (2017). Healthcare predictive analytics for risk profiling in chronic care: A bayesian multitask learning approach. MIS Quart..

[4] Gu D., Liang C., Zhao H. (2017). A case-based reasoning system based on weighted heterogeneous value distance metric for breast cancer diagnosis. Artif. Intell. Med..

[5] Wu C.-W., Shen H.-L., Lu C.-J., Chen S.-H., Chen H.-Y. (2021). Comparison of Different Machine Learning Classifiers for Glaucoma Diagnosis Based on Spectralis OCT. Diagnostics.

[6] Liu N., Qi E.S., Xu M., Gao B., Liu G.Q. (2019). A novel intelligent classification model for breast cancer diagnosis. Inf. Process. Manag..

[7] Liang H.Y., Tsui B.Y., Ni H., Valentim C.C.S., Baxter S.L., Liu G., Cai W., Kermany D.S., Sun X., Chen J. (2019). Evaluation and accurate diagnoses of pediatric diseases using artificial intelligence. Nat. Med..

[8] Koteluk O., Wartecki A., Mazurek S., Kołodziejczak I., Mackiewicz A. (2021). How do machines learn? Artificial intelligence as a new era in medicine. J. Pers. Med..

[9] Bardhan I., Oh J.-h., Zheng Z., Kirksey K. (2015). Predictive analytics for readmission of patients with congestive heart failure. Inform. Syst. Res..

[10] Zhou S.J., Li X. (2020). Feature engineering vs. deep learning for paper section identification: Toward applications in Chinese medical literature. Inf. Process. Manag..

[11] Hsu W.Y. (2018). A decision-making mechanism for assessing risk factor significance in cardiovascular diseases. Decis. Support Syst..

[12] Gu D.X., Su K.X., Zhao H.M. (2020). A case-based ensemble learning system for explainable breast cancer recurrence prediction. Artif. Intell. Med..

[13] Jussupow E., Spohrer K., Heinzl A., Gawlitza J. (2021). Augmenting medical diagnosis decisions? An investigation into physicians’ decision-making process with artificial intelligence. Inform. Syst. Res..

[14] Ahsen M.E., Ayvaci M.U.S., Raghunathan S. (2019). When algorithmic predictions use human-generated data: A bias-aware classification algorithm for breast cancer diagnosis. Inform. Syst. Res..

[15] Topol E.J. (2019). High-performance medicine: The convergence of human and artificial intelligence. Nat. Med..

[16] Chai Y., Bian Y., Liu H., Li J., Xu J. (2021). Glaucoma diagnosis in the Chinese context: An uncertainty information-centric Bayesian deep learning model. Inf. Process. Manag..

[17] Li S.Z., Jain A.K. (2009). Encyclopedia of Biometrics.

[18] Huang G., Song S., Gupta J.N.D., Wu C. (2014). Semi-supervised and unsupervised extreme learning machines. IEEE Trans. Cybern..

[19] Tseng C.J., Lu C.-J., Chang C.-C., Chen G.-D., Cheewakriangkrai C. (2017). Integration of data mining classification techniques and ensemble learning to identify risk factors and diagnose ovarian cancer recurrence. Artif. Intell. Med..

[20] Brown G., Wyatt J., Harris R., Yao X. (2005). Diversity creation methods: A survey and categorization. Inform. Fusion.

[21] Woźniak M., Graña M., Corchado E. (2014). A survey of multiple classifier systems as hybrid systems. Inform. Fusion.

[22] Das R., Turkoglu I., Sengur A. (2009). Effective diagnosis of heart disease through neural networks ensembles. Expert Syst. Appl..

[23] Al-Tashi Q., Rais H., Abdulkadir S.J. (2018). Hybrid swarm intelligence algorithms with ensemble machine learning for medical diagnosis. Proceedings of the 4th International Conference on Computer and Information Sciences.

[24] Zhou Z.H., Feng J. (2017). Deep forest: Towards an alternative to deep neural networks. Proceedings of the 26th International Joint Conference on Artificial Intelligence.

[25] Cao Y., Geddes T.A., Yang J.Y.H., Yang P.Y. (2020). Ensemble deep learning in bioinformatics. Nat. Mach. Intell..

[26] Bergstra J., Bardenet R., Bengio Y., Kégl B. (2011). Algorithms for hyper-parameter optimization. Proceedings of the 24th International Conference on Neural Information Processing Systems.

[27] Zhou Z.H. (2012). Ensemble Methods-Foundations and Algorithms.

[28] Cruz R.M.O., Sabourin R., Cavalcanti G.D.C. (2018). Dynamic classifier selection: Recent advances and perspectives. Inform. Fusion.

[29] Chandra A., Xin Y. (2004). DIVACE: Diverse and accurate ensemble learning algorithm. Proceedings of the International Conference on Intelligent Data Engineering and Automated Learning.

[30] Britto A.S., Sabourin R., Oliveira L.E.S. (2014). Dynamic selection of classifiers—A comprehensive review. Pattern Recogn..

[31] Brun A.L., Britto A.S., Oliveira L.S., Enembreck F., Sabourin R. (2018). A framework for dynamic classifier selection oriented by the classification problem difficulty. Pattern Recogn..

[32] Junior L.M., Nardini F.M., Renso C., Trani R., Macedo J.A. (2020). A novel approach to define the local region of dynamic selection techniques in imbalanced credit scoring problems. Expert Syst. Appl..

[33] Ekbal A., Saha S. (2011). A multiobjective simulated annealing approach for classifier ensemble: Named entity recognition in Indian languages as case studies. Expert Syst. Appl..

[34] García-Gutiérrez J., Mateos-García D., Garcia M., Riquelme-Santos J.C. (2015). An evolutionary-weighted majority voting and support vector machines applied to contextual classification of LiDAR and imagery data fusion. Neurocomputing..

[35] Snoek J., Larochelle H., Adams R.P. (2012). Practical Bayesian Optimization of Machine Learning Algorithms. Proceedings of the 25th International Conference on Neural Information Processing Systems.

[36] Hernández-Lobato J.M., Hoffman M.W., Ghahramani Z. (2014). Predictive entropy search for efficient global optimization of black-box functions. Neural Inform. Process. Syst..

[37] Ilievski I., Akhtar T., Feng J., Shoemaker C.A. (2017). Efficient hyperparameter optimization for deep learning algorithms using deterministic rbf surrogates. Proceedings of the 31th AAAI Conference on Artificial Intelligence.

[38] Zhang M., Li H., Pan S., Lyu J., Ling S., Su S. (2021). Convolutional neural networks-based lung nodule classification: A surrogate-assisted evolutionary algorithm for hyperparameter optimization. IEEE Trans. Evol. Comput..

[39] Pérez-Gállego P., Castaño A., Quevedo J.R., Coz J.J.D. (2018). Dynamic ensemble selection for quantification tasks. Inform. Fusion.

[40] Wolpert D.H. (1992). Stacked generalization. Neural Netw..

[41] Dongdong L., Ziqiu C., Bolu W., Zhe W., Hai Y., Wenli D. (2021). Entropy-based hybrid sampling ensemble learning for imbalanced data. Int. J. Intell. Syst..

[42] Xu S.J., Pan Z.G. (2020). A novel ensemble of random forest for assisting diagnosis of Parkinson’s disease on small handwritten dynamics dataset. Int. J. Med. Inform..

[43] Geurts P., Ernst D., Wehenkel L. (2006). Extremely randomized trees. Mach. Learn..

[44] Friedman J.H. (1999). Greedy function approximation: A gradient boosting machine. Ann. Stat..

[45] Breiman L. (2001). Random Forests. Mach. Learn..

[46] Scornet E. (2016). Random Forests and Kernel methods. IEEE Trans. Inform. Theory.

[47] Kononenko I. (2001). Machine learning for medical diagnosis: History, state of the art and perspective. Artif. Intell. Med..

[48] Alizadehsani R., Habibi J., Hosseini M.J., Mashayekhi H., Boghrati R., Ghandeharioun A., Bahadorian B., Sani Z.A. (2013). A data mining approach for diagnosis of coronary artery disease. Comput. Meth. Prog. Biomed..

[49] Dua D., Graff C. (2017). UCI Machine Learning Repository.

[50] Fernandes K., Cardoso J.S., Fernandes J. (2017). Transfer learning with partial observability applied to cervical cancer screening. Pattern Recognition and Image Analysis. IbPRIA.

[51] Shabani-Mashcool S., Marashi S.-A., Gharaghani S. (2020). NDDSA: A network- and domain-based method for predicting drug-side effect associations. Inf. Process. Manag..

[52] Park S.H., Han K. (2018). Methodologic guide for evaluating clinical performance and effect of artificial intelligence technology for medical siagnosis and prediction. Radiology.

[53] Swami A., Jain R. (2012). Scikit-learn: Machine learning in Python. J. Mach. Learn. Res..

[54] Bergstra J., Yamins D., Cox D.D. Making a science of model search: Hyperparameter optimization in hundreds of dimensions for vision architectures. Proceedings of the 30th International Conference on International Conference on Machine Learning.

[55] Angst C.M., Wowak K.D., Handley S.M., Kelley K. (2017). Antecedents of information systems sourcing strategies in US hospitals: A longitudinal study. MIS Quart..

[56] Diao X.L., Huo Y.N., Zhao S., Yuan J., Cui M., Wang Y.X., Lian X.D., Zhao W. (2021). Automated ICD coding for primary diagnosis via clinically interpretable machine learning. Int. J. Med. Inform..

[57] Gu D., Zhao W., Xie Y., Wang X., Su K., Zolotarev O.V. (2021). A Personalized Medical Decision Support System Based on Explainable Machine Learning Algorithms and ECC Features: Data from the Real World. Diagnostics.

